# Explainable deep learning reveals a stagewise temporal deterioration from skin tone to contour to texture in Chinese women’s skin aging

**DOI:** 10.3389/fmed.2026.1945376

**Published:** 2026-09-25

**Authors:** Guo-long Dong, Fan Hu, Ya-wen Liu, Wei-zhen Xiao, Fan Yi, Chen-juan Zhang, Rui-min Liu, Yue Wu

**Affiliations:** 1 School of Light Industry and Chemical Engineering, Beijing Technology and Business University, Beijing, China; 2 Institute of Cosmetic Regulatory Science, Beijing Technology and Business University, Bejing, China; 3 Corporate R&D Department, Shanghai Pechoin Daily Chemical Co., Ltd., Shanghai, China

**Keywords:** aging stage classification, facial imaging, multimodal deep learning, shapley additive explanations, skin aging

## Abstract

Skin aging is a continuous and progressive physiological process characterized by gradual structural and functional changes. Early identification and accurate assessment of aging-related facial features are essential for developing targeted skincare strategies. This study aimed to establish a deep learning-based framework combined with Shapley Additive Explanations (SHAP) analysis to classify facial aging stages in Chinese women and provide interpretable evidence for personalized skincare. A total of 300 Chinese women aged 18–60 years were included. Standardized facial images were acquired using the VISIA imaging system. Four dimensions, including tone, contour, texture, and porphyrin, were evaluated, and 23 facial indicators were extracted. A hybrid multimodal deep learning classification model was developed by integrating facial image data with quantitative facial aging indicators. Five-fold stratified cross-validation was performed to evaluate model performance, with Top-2 accuracy used as the primary evaluation metric. SHAP analysis was further applied to assess the contribution weights of different facial aging indicators to model predictions. Based on the fitted quantitative facial aging indicators, a six-class facial aging classification model was established. The model demonstrated stable convergence and achieved a highest validation Top-2 accuracy of 85.0%. The confusion matrix showed that classification errors occurred primarily between adjacent aging stages. SHAP analysis revealed a progressive aging pattern characterized by early changes in skin color, middle-stage changes in facial contour, and later deterioration in skin texture. This study demonstrates that integrating deep learning with SHAP analysis can effectively classify facial aging stages in Chinese women while providing interpretable insights into the key features driving aging-related predictions.

## 1 Introduction

Facial skin aging arises from the combined effects of intrinsic biological aging and cumulative extrinsic exposures, particularly chronic ultraviolet exposure, and is clinically characterized by wrinkles, pigmentation abnormalities, uneven skin tone, surface roughness, enlarged pores, facial contour changes, and an overall decline in skin-surface quality (1–3). At the dermal level, intrinsic aging is closely associated with fibroblast senescence and dysfunction, leading to impaired collagen synthesis and disruption of extracellular matrix (ECM) homeostasis (4, 5). Age-related collagen fragmentation and altered fibroblast–ECM interactions further impair mechanical signaling and promote dermal structural deterioration (4, 6).

Previous studies have described several population-specific aging characteristics in Chinese women. Flament et al. (7) evaluated facial clinical signs and perceived age in Chinese women and reported that wrinkles, pigmentation disorders, sagging, and texture-related changes were closely associated with age perception. Porcheron et al. (8) further demonstrated that the perceptual contributions of specific aging signs differ between Chinese and Caucasian faces. Zhang et al. (9) analyzed skin phenotypes across multiple facial regions in Chinese women and proposed potential transitional periods during aging, whereas Quan et al. (10) identified skin features associated with an older perceived age in women aged 20–40 years.

Flament et al. (11) applied artificial intelligence to analyze more than half a million facial images from European and Chinese women, revealing population-specific trajectories of wrinkles, pigmentation, pores, vascular signs, and sagging. Collectively, these studies have shifted facial aging research from the observation of individual signs toward comprehensive phenotypic profiling. However, most remain descriptive, correlation-based, or dependent on chronological age grouping and therefore do not fully explain how multiple quantitative indicators jointly contribute to aging-stage classification.

Deep learning (DL) provides a useful approach for extracting complex visual information from facial images. Convolutional neural networks (CNNs) are widely used in image recognition because they can learn hierarchical visual features directly from raw images (12, 13). CNN-based models have also been applied to dermatological classification tasks. Gessert et al. (14) used multi-resolution EfficientNets combined with metadata for skin lesion classification, while Manole et al. (15) developed an EfficientNetB3-based model to classify multiple dermatological conditions. In facial skin assessment, Watanabe et al. (16) developed a deep learning model for acne severity classification using facial images. These studies support the feasibility of using CNN-derived image features for skin-related classification tasks; however, similar approaches remain limited in facial aging-stage classification.

Multimodal learning generally refers to the integration of heterogeneous data sources, including medical images, clinical variables, laboratory measurements, electronic health records, textual information, and other tabular data, within a unified predictive model (17, 18). Model interpretability is also essential because facial aging assessment should identify not only the predicted aging stage but also the phenotypic basis underlying the prediction. SHAP analysis is a feature-attribution method for supervised machine learning models that quantifies the contributions of individual variables to both local predictions and global feature-importance patterns (19–21).

Our previous studies established a basis for phenotype-based facial aging assessment in Chinese women by characterizing wrinkle-related aging stages and developing a five-dimensional skin color evaluation system (22, 23). Previous facial aging studies have mainly focused on individual phenotypic signs or predefined age groups, whereas AI-based studies have primarily emphasized image-based prediction, with limited integration of quantitative regional facial features. Therefore, a gap remains in combining multidimensional facial phenotypes with image-based information for interpretable aging-stage classification. The present study integrated age-related trajectories of facial tone, contour, texture, and porphyrin with whole-face images in a multimodal framework, while complementary feature-attribution and image-visualization analyses were used to interpret aging-stage predictions.

## 2 Materials and methods

The overall experimental workflow is shown in Figure 1.

**Figure 1:**
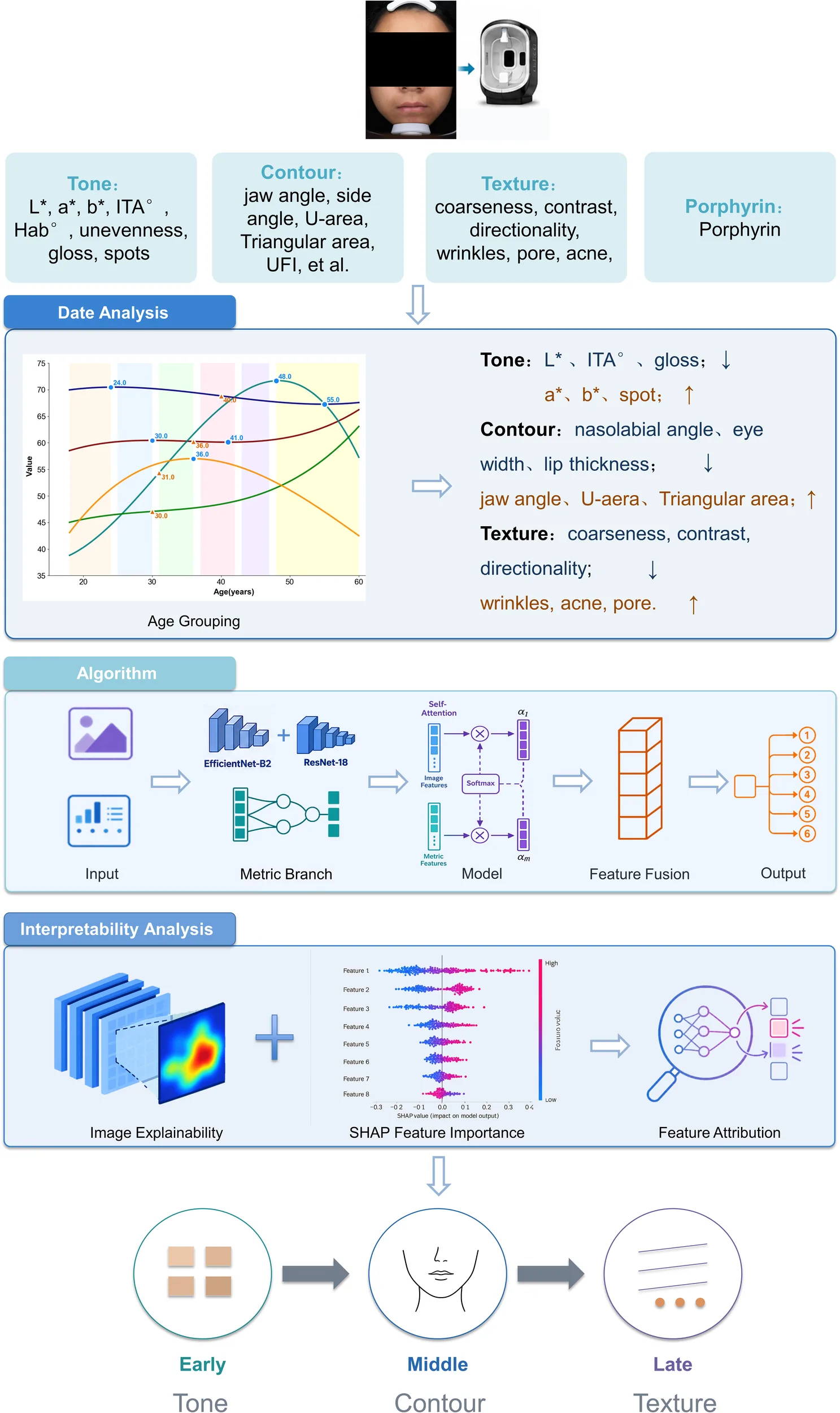
Overall workflow of the facial aging classification model.

### 2.1 Volunteer recruitment

The study was conducted in strict accordance with the principles outlined in the Declaration of Helsinki. A total of 300 Chinese female volunteers aged 18–60 years were screened and selected from the database according to the inclusion criteria. To ensure a balanced age distribution, participants were enrolled across 43 age groups spanning 18 to 60 years. Each age group included 7 volunteers, except for the 60-year-old group, which included 6 volunteers. The mean age of the participants was 38.93 years, with a standard deviation of 12.39 years. Chronological age was used as the reference variable for all age-related analyses and for assigning participants to the six aging-stage categories.

Participants were screened using predefined inclusion and exclusion criteria to minimize potential confounding from major skin conditions, recent cosmetic interventions, and occupational sun exposure. Most participants had predominantly indoor occupations, thereby limiting the range of long-term cumulative UV exposure within the study population.

All respondents from Beijing (39°56′N, 116°20′E), Shanghai (31°22′N, 121°48′E), Wuxi (31°57′N, 120°30′E), Wuhan (30°52′N, 114°31′E), and Taiyuan (37°87′N, 112°53′E) had resided in their respective locations for at least 12 months.

### 2.2 Facial aging features extraction

#### 2.2.1 Skin contour features extraction

Conventional facial contour parameters were extracted from standardized frontal facial images using an 81-point facial landmark detection model implemented with the dlib library. Based on the corresponding landmark coordinates, five contour related parameters—jaw angle, side angle, lower face width, nasolabial angle, and nasal root angle—were calculated to characterize age-related changes in lower facial shape, facial width, and the geometric relationships among facial structures. The definitions and measurement procedures for these contour parameters are illustrated in Figures 2A–E.

**Figure 2:**
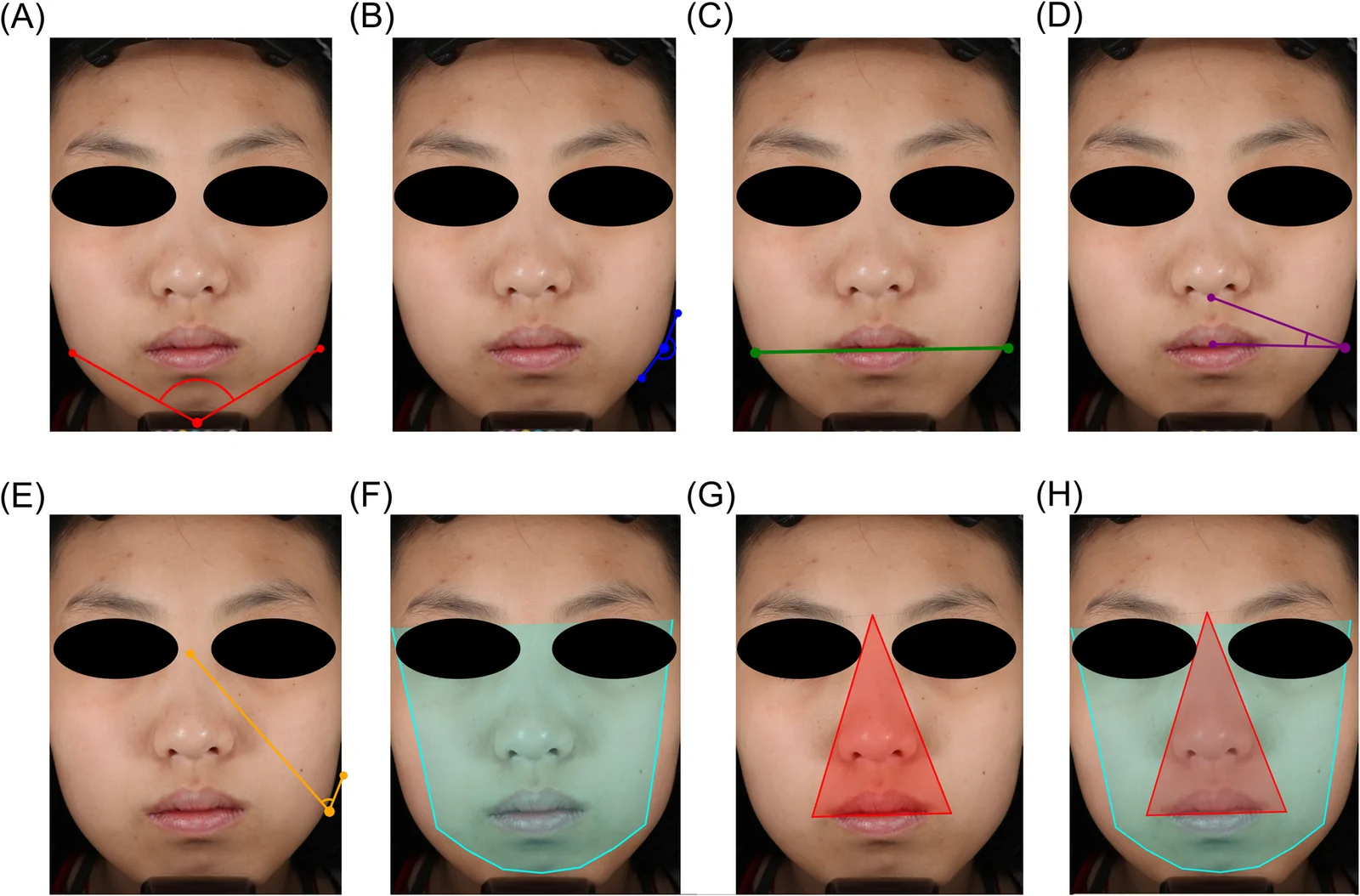
Schematic illustration of facial contour measurements and the UFI model. **(A)** Jaw angle; **(B)** side angle; **(C)** lower face width; **(D)** nasolabial angle; **(E)** nasal root angle; **(F)** U-area; **(G)** triangular area; and **(H)** UFI.

Facial contour parameters were calculated based on the coordinates of detected facial landmarks. For angular measurements (jaw angle, side angle, nasolabial angle, and nasal root angle), the included angle formed by three corresponding landmarks was calculated using the cosine formula (Equation 1).

$$\theta =\mathrm{co}s^{-1}\frac{\left(A-B\right)\cdot \left(C-B\right)}{|A-B||C-B|}$$ (1)

where A, B, and C represent the coordinates of the three facial landmarks defining each angle.

Lower face width was calculated as the Euclidean distance between two predefined landmarks (Equation 2).

$$D=\sqrt{{\left(x_{2}-x_{1}\right)}^{2}+{\left(y_{2}-y_{1}\right)}^{2}}$$ (2)

where (x_1_,y_1_) and (x_2_,y_2_) represent the coordinates of the two landmarks. All distances were measured in pixel units.

To quantitatively characterize age-related changes in facial contour morphology, a U-frame Index (UFI) was constructed using geometric features derived from facial landmark coordinates (Figures 2F–H).

Facial landmarks were detected using an 81-point facial landmark model, and contour-related regions were defined based on anatomically relevant facial reference points. Previous anatomical and three-dimensional morphometric studies have demonstrated that facial aging is associated with progressive skeletal remodeling, redistribution of facial volume, soft tissue descent, and alterations in lower facial width and contour definition (24, 25). Age-related redistribution of facial volume and downward displacement of facial soft tissues contribute to reduced facial tapering and increased lower facial heaviness, which are commonly characterized as a shift from a youthful inverted-triangle configuration toward a broader lower facial contour (24, 26). Based on these established morphological characteristics, the lower facial U-shaped region and the central triangular region were selected as representative geometric components to quantify age-associated contour remodeling. The boundaries of these regions were defined by facial landmark connections corresponding to the lower facial contour and midfacial reference points (Figures 2F–H).

#### 2.2.2 Features extraction in tone and texture dimension

Facial images of 300 Chinese female volunteers aged 18–60 years were selected from the database and included in this study. All facial images were acquired using the VISIA imaging system under standardized environmental conditions to ensure consistency and reproducibility. These four categories were selected based on their relevance to age related facial changes. Tone-related parameters reflect alterations in skin color and pigmentation, contour related parameters capture age associated facial morphological remodeling, and texture related parameters represent surface changes such as wrinkles and pores. Porphyrin parameters were included as complementary indicators of skin surface characteristics to provide a more comprehensive assessment of facial aging. Accordingly, aging related facial features, including tone, contour, texture, and porphyrin indicators, were extracted using PyCharm software (Table 1) and organized as structured variables for subsequent model development.

**Table 1:** Facial feature indicators.

Dimension	Indicator
Tone	L*, a*, b*, ITA°, Hab°, unevenness, gloss, spots (total number)
Contour	jaw angle, side angle, lower face width, nasolabial angle, nasal root angle, left eye width, right eye width, lip thickness, U-area, Triangular area, UFI
Texture	coarseness, contrast, directionality, wrinkles (total wrinkles number), acne, pore (number)
Porphyrin	Porphyrin (number)

### 2.3 Statistical analysis

Statistical analyses were performed to investigate the associations between chronological age and facial aging indicators derived from VISIA images. Continuous variables are presented as the mean ± standard deviation for normally distributed data or as the median and interquartile range for non-normally distributed data. Data normality was assessed using the Shapiro–Wilk test. Differences in facial aging indicators among age groups were evaluated using one-way analysis of variance (ANOVA) or the Kruskal–Wallis test, depending on the data distribution. When significant between-group differences were identified, *post hoc* multiple comparisons were performed using Tukey's test following ANOVA or Dunn's test with Bonferroni correction following the Kruskal–Wallis test.

Chronological age was used as the independent variable, and the facial feature indicators listed in Table 1 were used as dependent variables for age-related trend fitting. Polynomial fitting was performed to characterize the nonlinear trajectories of these indicators with increasing age.

To determine the optimal polynomial model for describing age-related changes in facial indicators, linear, quadratic, and cubic polynomial regression models were compared (Supplementary Table S1). Model selection was based on multiple statistical criteria, including adjusted *R*^2^, Akaike information criterion (AIC), Bayesian information criterion (BIC), and the significance of polynomial terms.

All statistical analyses were performed using SPSS Statistics version 27.0 (IBM Corp., Armonk, NY, USA) and Python version 3.9. All tests were two-sided, and a *p*-value < 0.05 was considered statistically significant.

### 2.4 Deep learning model construction

#### 2.4.1 Dataset preparation, preprocessing, and augmentation

Before model training, all facial images underwent standardized preprocessing to minimize variations in image size, noise, and facial position. The images were resized to 768 × 512 pixels, aligned and denoised, and subsequently normalized using the mean and standard deviation of the ImageNet dataset.

To improve model generalization and reduce the risk of overfitting, data augmentation was applied exclusively to the training images. The augmentation strategies included random rotation, scaling, translation, horizontal and vertical flipping, brightness and contrast adjustment, hue–saturation–value transformation, optical distortion, grid distortion, random cropping, Gaussian noise, Gaussian blur, gamma correction, sharpening, and random sun-flare simulation. For the validation images, only resizing, normalization, and tensor conversion were performed.

Stratified five-fold cross-validation was used to evaluate model robustness. In each fold, approximately 80% of the samples were used for model training, while the remaining 20% were used for validation. A comparable class distribution was maintained across all five folds.

#### 2.4.2 Hybrid multimodal model architecture

A hybrid multimodal deep learning model was developed to classify the six facial age groups by integrating facial images with quantitative facial aging indicators. The overall architecture of the proposed model is shown in Figure 3.

**Figure 3:**
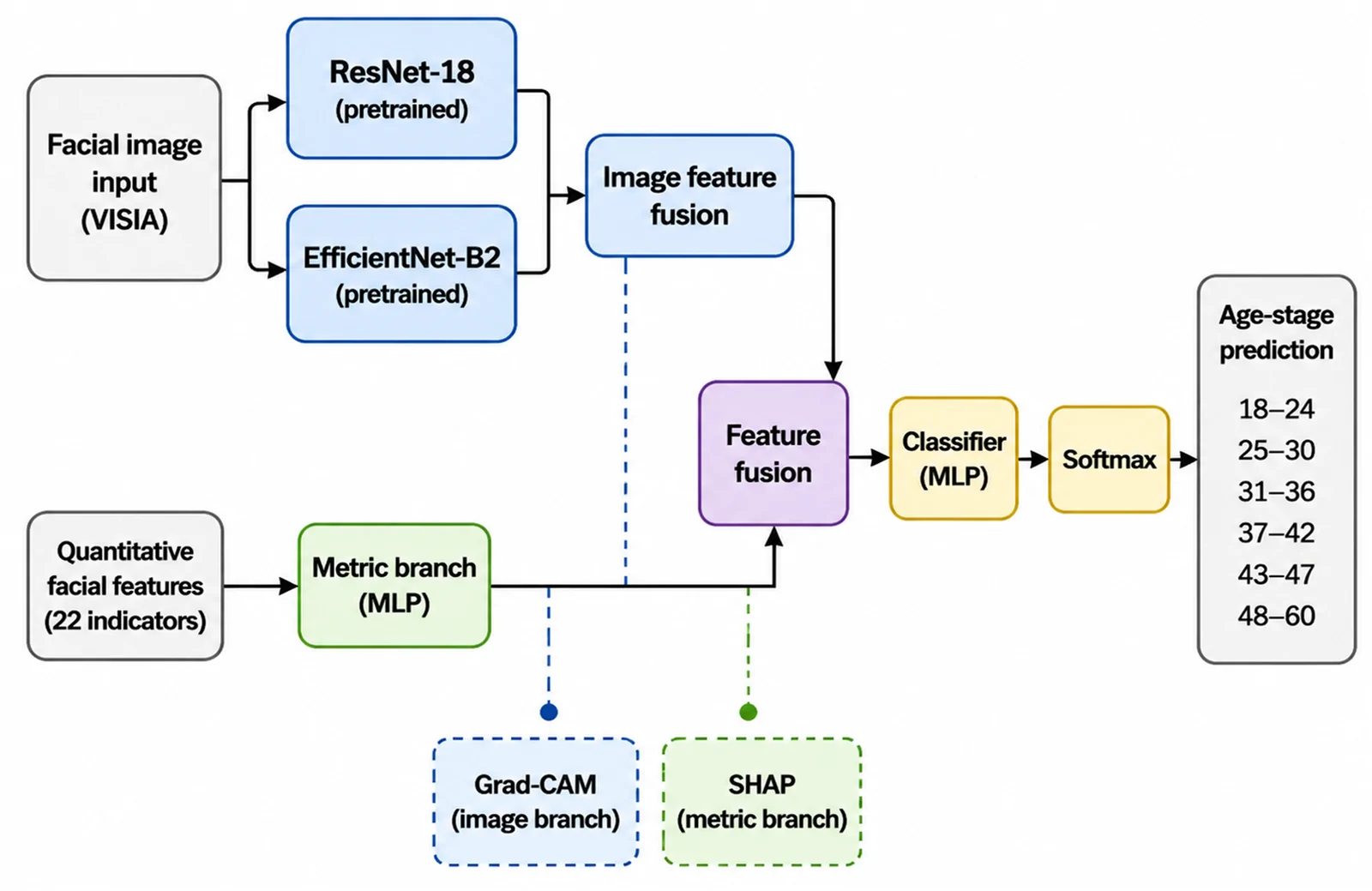
Architecture of the proposed hybrid multimodal deep learning model for six-class facial age-group classification.

The image branch comprised EfficientNet-B2 and ResNet-18 networks pretrained on the ImageNet dataset. The final classification layers of both networks were removed, allowing the two networks to serve as parallel image feature extractors. The extracted features were then concatenated and compressed through fully connected layers incorporating batch normalization, GELU activation, and dropout.

In parallel, the selected quantitative facial aging indicators were standardized and fed into a multilayer perceptron branch. Porphyrin-related features were excluded from the model input because they were extracted from images acquired under ultraviolet illumination rather than from standardized facial photographs. The image-derived features and quantitative indicator-derived features were subsequently integrated through an attention-based fusion module. The fused representation was then passed through a multilayer classifier to generate predicted probabilities for the six age groups.

#### 2.4.3 Evaluation metrics

The model was trained for 100 epochs in each fold using the AdamW optimizer. Weighted cross-entropy loss and a weighted random sampler were employed to mitigate the effects of class imbalance. Dropout and weight decay were applied as regularization strategies. Training and validation losses were recorded at each epoch to monitor model convergence and potential overfitting.

Model performance was evaluated using classification accuracy, weighted F1-score, and Top-2 accuracy. Top-2 accuracy was defined as the proportion of samples for which the true class was among the two classes with the highest predicted probabilities. Confusion matrices were also generated to evaluate class-specific recognition performance and the distribution of misclassifications across the six age groups.

### 2.5 Interpretability analysis

To improve the interpretability of the proposed multimodal deep learning framework, complementary explainability approaches were applied to characterize the contribution of both image-based and quantitative facial features.

For the image modality, Gradient-weighted Class Activation Mapping (Grad-CAM) was applied directly to the trained convolutional neural network branches (EfficientNet-B2 and ResNet-18) to visualize facial regions associated with the predicted age stage. The generated activation maps highlight spatial regions that contributed most strongly to the model prediction, providing an intuitive interpretation of image-derived features.

For the quantitative facial feature modality, Kernel SHapley Additive exPlanations (Kernel SHAP) was applied within the trained multimodal framework rather than an independent surrogate classifier. Specifically, for each participant, the image embedding extracted from the trained image branches was fixed, while the standardized quantitative facial indicators were perturbed and propagated through the original metric branch and fusion classifier. Therefore, SHAP values represent the contribution of individual facial measurements to the prediction output under the context of the actual multimodal model.

By combining Grad-CAM and conditional SHAP analysis, the proposed framework provides interpretation from complementary perspectives: Grad-CAM identifies spatial facial characteristics learned from images, whereas SHAP quantifies the contribution of measurable facial aging indicators. This approach enables a more comprehensive understanding of how heterogeneous facial information contributes to age-stage classification.

## 3 Results

### 3.1 Descriptive statistics of quantitative facial indicators

Quantitative facial indicators were extracted to characterize facial skin status and aging-related phenotypes in Chinese female volunteers. These indicators were categorized into four groups: tone, contour, texture, and porphyrin. Descriptive statistical analyses were performed for all extracted skin parameters, and the results are summarized in Table 2. These quantitative indicators provided a structured characterization of facial features and were subsequently used for age-related trend analysis and model construction.

**Table 2:** Descriptive statistics of whole-face skin feature indicators.

Dimension	Indicator	Mean ± SD
Tone	L*	63.52 ± 3.18
	a*	17.83 ± 2.13
	b*	20.42 ± 2.31
	ITA°	31.05 ± 8.12
	Hab°	48.96 ± 3.85
	unevenness	33.63 ± 2.84
	Highlight	0.09 ± 0.06
	Spots	89.27 ± 44.26
Contour	Jaw angle	107.68 ± 4.76
	Side angle	168.10 ± 1.60
	Lower face width	2,687.00 ± 126.50
	Nasolabial angle	19.85 ± 1.74
	Nasal root angle	61.44 ± 3.66
	left eye width	634.12 ± 30.27
	right eye width	628.43 ± 30.87
	Lip thickness	201.74 ± 14.07
	U-area	31.69 ± 1.33
	Triangular area	102.61 ± 5.43
Texture	coarseness	26.89 ± 0.56
	contrast	60.82 ± 5.00
	directionality	0.67 ± 0.01
	wrinkles	4.88 ± 3.56
	Acne	13.81 ± 9.61
	Pore	652.49 ± 645.84
Porphyrin	porphyrin	2,220.05 ± 4,882.83

### 3.2 Age-related fitting of facial aging indicators

Polynomial fitting was performed for each indicator to characterize age-related trends (Table 3 and Figure 4). Within the skin tone dimension, L* and ITA° values peaked at 22–26 years of age and gradually declined thereafter, indicating progressive skin darkening. The b* value decreased slightly during early adulthood and increased steadily after 29 years of age, whereas the a* value gradually increased between 20 and 47 years, reflecting age-dependent changes in skin redness. Hab° and unevenness progressively increased, with inflection points occurring at approximately 35–36 years of age, indicating increasing skin color irregularity and age-related chromatic changes. Gloss increased slowly with age, while the number of pigmented spots showed a continuous upward trend and peaked at approximately 48 years of age.

**Table 3:** Polynomial fitting results of facial skin indicators across four dimensions, with stationary and inflection points.

Dimension	Indicator	Polynomial ftting curve formula	Stationary point 1	Stationary point 2	Inflection point	*R* ^2^
Tone	L*	y = 0.000312x^3^ − 0.037001x^2^ + 1.279354x + 51.453319	25.55	53.46	39.50	0.56
	a*	y = −0.000220x^3^ + 0.022122x^2^ − 0.627171x + 22.268370	20.38	46.55	33.47	0.44
	b*	y = −0.000086x^3^ + 0.007628x^2^ − 0.108178x + 18.528390	—	50.56	29.40	0.69
	ITA°	y = 0.000693x^3^ − 0.078477x^2^ + 2.491344x + 11.744886	22.70	52.80	37.75	0.65
	Hab°	y = 0.000239x^3^ − 0.025431x^2^ + 0.879258x + 38.783239	29.82	41.13	35.48	0.31
	Unevenness	y = 0.000049x^3^ − 0.010096x^2^ + 0.540228x + 25.708658	—	36.42	—	0.22
	Gloss	y = 0.000006x^3^ − 0.000751x^2^ + 0.028734x − 0.219778	28.70	57.40	43.10	0.61
	Spots	y = −0.002859x^3^ + 0.267267x^2^ − 5.793102x + 88.325578	—	48.36	31.16	0.65
Contour	Jaw angle	y = −0.000111x^3^ + 0.019736x^2^ − 0.957735x + 120.555370	—	34.00	59.40	0.26
	Side angle	y = 0.000051x^3^ − 0.007935x^2^ + 0.359855x + 163.398857	—	33.60	51.70	0.21
	Lower face width	y = −0.008483x^3^ + 1.182273x^2^ − 50.725430x + 3,341.307421	33.60	59.30	46.50	0.23
	Nasolabial angle	y = −0.000051x^3^ + 0.003910x^2^ − 0.075290x + 20.173164	—	38.40	25.60	0.27
	Nasal root angle	y = 0.000116x^3^ − 0.013819x^2^ + 0.469070x + 57.337771	24.50	55.20	39.80	0.14
	Left eye width	y = −0.002147x^3^ + 0.273762x^2^ − 11.761219x + 800.227553	—	—	42.51	0.50
	Right eye width	y = −0.001827x^3^+ 0.249758x^2^ − 11.363270x + 794.562417	43.83	47.29	45.56	0.53
	Lip thickness	y = −0.000463x^3^ + 0.054434x^2^ − 2.448697x + 241.842414	—	—	39.21	0.54
	U-area	y = 0.000023x^3^ − 0.000279x^2^ − 0.033440x + 102.531384	26.24	—	—	0.46
	Triangular area	y = 0.000061x^3^ − 0.007190x^2^ + 0.286965x + 27.839947	—	—	39.41	0.65
Texture	Coarseness	y = −0.000015x^3^ + 0.000811x^2^ − 0.016597x + 27.356788	—	—	—	0.81
	Contrast	y = 0.000271x^3^ − 0.042914x^2^ + 1.881426x + 38.326975	31.10	—	52.80	0.71
	Directionality	y = −0.0000000382x^3^ + 0.00000304x^2^ − 0.000203x + 0.672443	—	—	26.53	0.28
	Wrinkles	y = 0.000254x^3^ − 0.023112x^2^ + 0.746663x − 5.079793	—	—	30.40	0.82
	Acne	y = −0.000476x^3^ + 0.063256x^2^ − 2.788156x + 53.213569	40.99	47.67	44.33	0.37
	Pore	y = −0.006834x^3^ + 1.802222x^2^ − 83.102911x + 1,406.593043	27.30	—	—	0.73
Porphyrin	Porphyrin	y = 0.018438x^3^ − 3.414394x^2^ + 173.505759x − 582.840938	—	35.77	—	0.10

**Figure 4:**
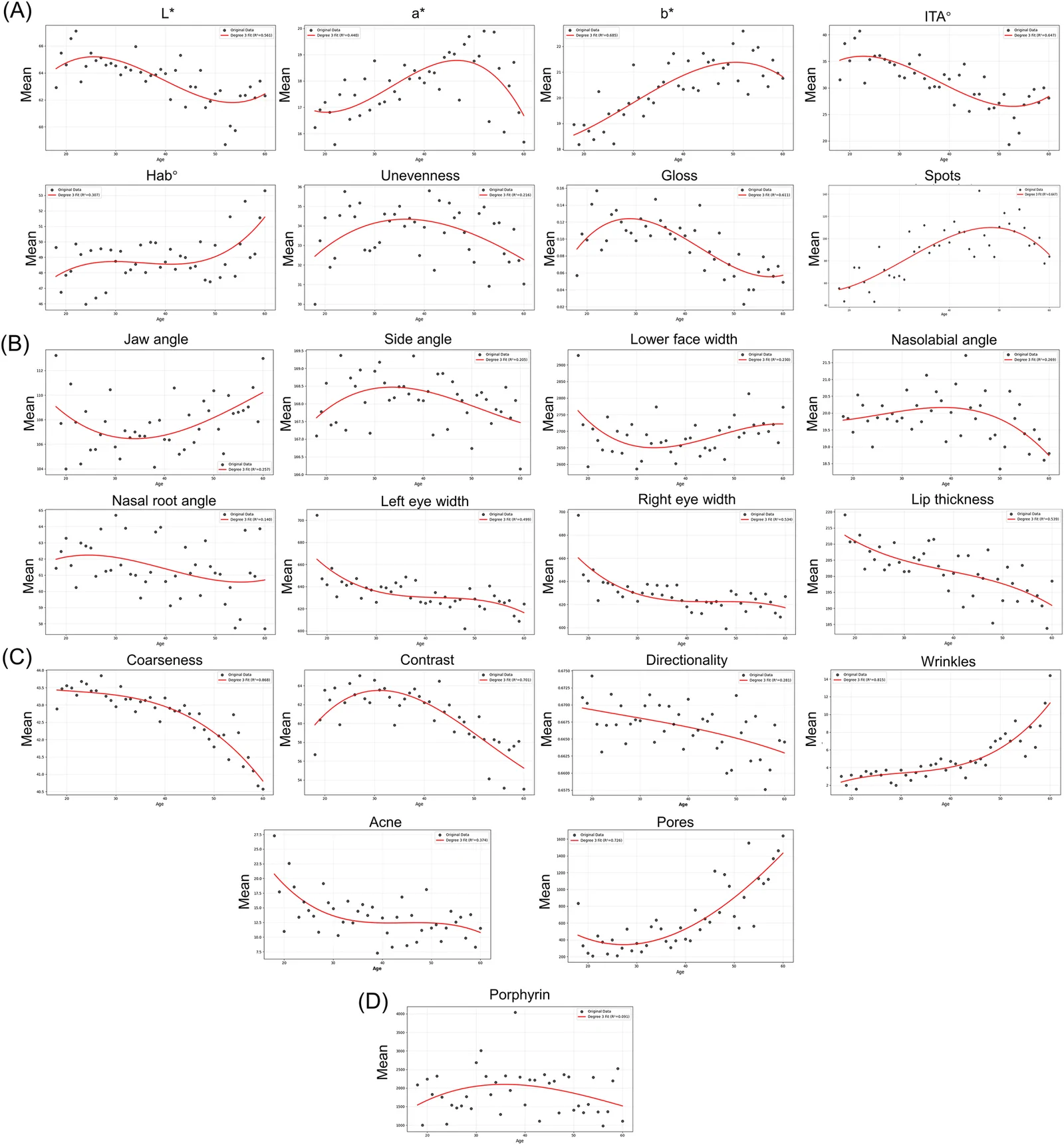
Age-dependent trends of facial indicators across four categories: **(A)** tone, **(B)** contour, **(C)** texture, and **(D)** porphyrin.

Within the facial contour dimension, jaw angle, side angle, lower face width, nasolabial angle, nasal root angle, eye width, and lip thickness exhibited slow but continuous age-dependent structural changes. Most parameters remained relatively stable before 30 years of age, followed by gradual changes between 31 and 42 years and more pronounced remodeling between 43 and 60 years, reflecting cumulative alterations in facial morphology.

Texture-related indicators, including coarseness, contrast, directionality, wrinkles, acne, and pores, exhibited nonlinear age-related trajectories. Wrinkle and pore counts increased steadily after the late 30s, with accelerated changes occurring between approximately 38 and 50 years of age. Acne prevalence gradually decreased with age, whereas coarseness and contrast increased, indicating progressive deterioration in skin microstructure and texture. Porphyrin levels also gradually increased throughout adulthood, with a noticeable rise after 36 years of age, suggesting cumulative physiological or microbial changes. Furthermore, uncertainty analysis was performed to evaluate the reliability of the estimated age-related transition points. The 95% confidence intervals of the stationary points and inflection points derived from the cubic polynomial models were calculated and are provided in Supplementary Table S2.

### 3.3 Age-related changes in facial contour based on UFI curves

The UFI model was established by combining the U-frame area and triangular area to characterize age-related changes in facial contour morphology (Figure 5). Age-dependent fitting analysis revealed distinct variation patterns for the two geometric parameters. The U-frame area remained relatively stable during early adulthood and subsequently increased gradually with advancing age, particularly after 40 years of age, indicating progressive alterations in the spatial structure of the lower facial contour. In contrast, the triangular area showed an overall increasing trend throughout the aging process, with a relatively rapid increase at younger ages, followed by a period of slower change and a subsequent increase in older individuals.

**Figure 5:**
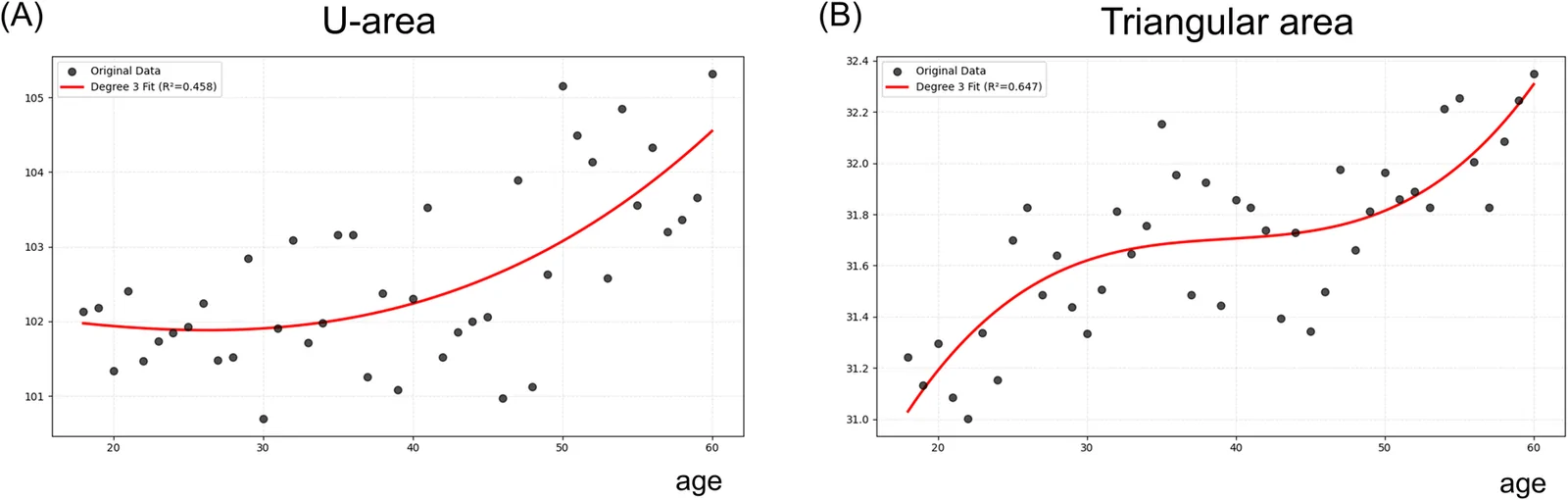
Age-fitting curves of the U-frame area and triangular area. **(A)**, U-Frame; **(B)**, Triangular index.

### 3.4 Establishment of six aging stages based on age-related feature fitting

Considering the four dimensions of facial skin indicators—tone, contour, texture, and porphyrin—together with their age-dependent trends, key stationary and inflection points derived from polynomial fitting were used to identify critical transitions in skin physiology and facial morphology. Representative indicators were selected to capture the features most sensitive to age-related changes throughout adulthood. By analyzing the fitted curves and identifying their stationary and inflection points, a dynamic segmentation of facial skin aging was established (Figure 6), providing a quantitative basis for characterizing facial skin changes in Chinese women across different age groups.

**Figure 6:**
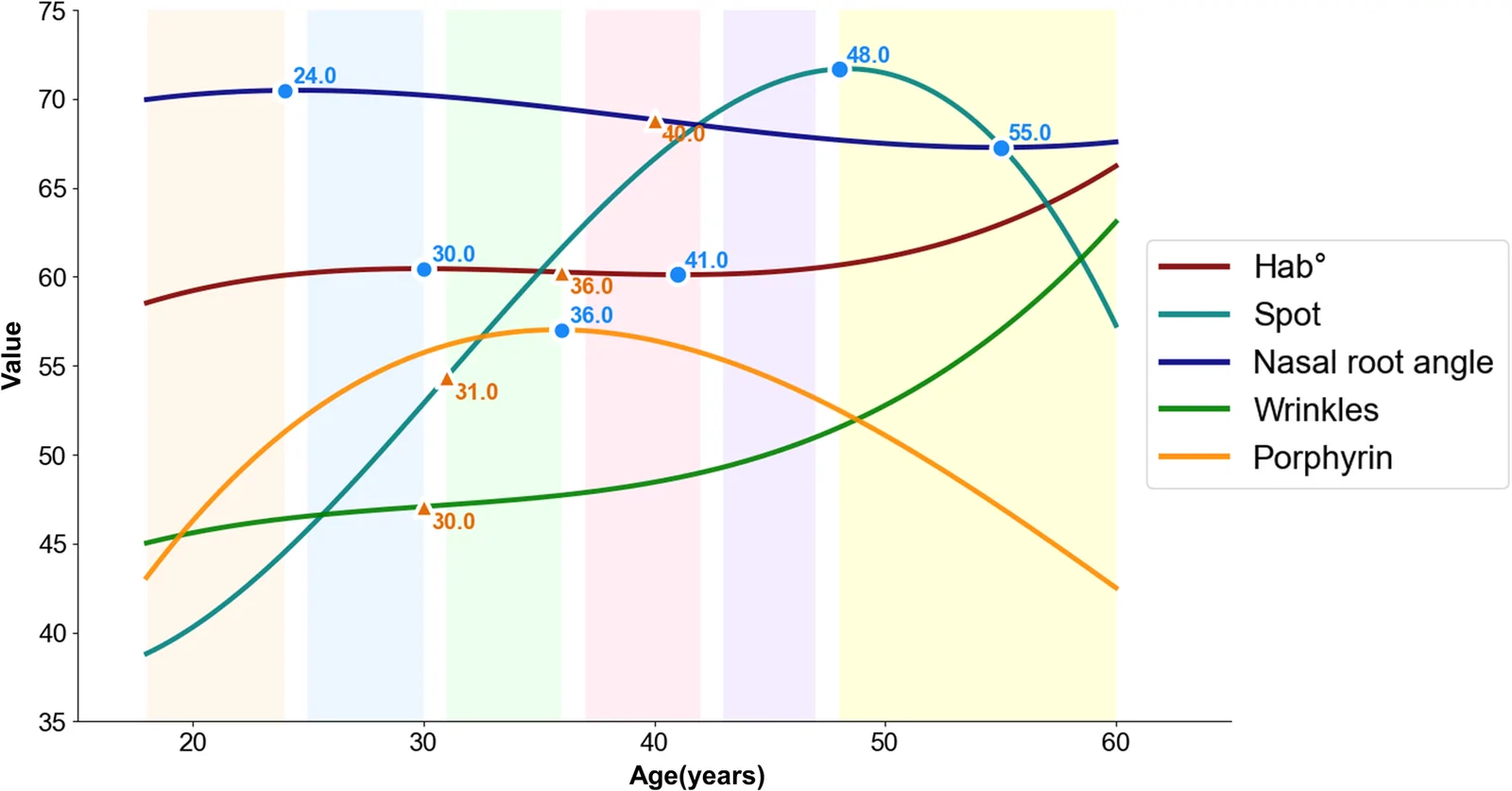
Dynamic segmentation of facial skin changes with age in Chinese women. (1) A1 (18–24 years); (2) A2 (25–30 years); (3) A3 (31–36 years); (4) A4 (37–42 years); (5) A5 (43–47 years); (6) A6 (48–60 years).

### 3.5 Differences in facial aging indicators across the six aging stages

Differences in facial aging indicators among the six age groups were evaluated using one-way analysis of variance (ANOVA) or the Kruskal–Wallis test, depending on the data distribution. As shown in Table 4 and Figure 7, significant intergroup differences were observed for most facial indicators, with relatively clear age-related variations in skin tone, facial contour, and skin texture.

**Table 4:** Descriptive statistics and intergroup comparison results of facial aging indicators across the six age groups.

Dimension	Indicator	A1	A2	A3	A4	A5	A6	P
Tone	L*	64.93 ± 2.77	64.89 ± 2.58	64.32 ± 3.25	63.68 ± 0.94	63.38 ± 0.88	61.72 ± 3.29	<0.0001
	a*	16.89 ± 1.70	17.41 ± 1.86	17.65 ± 1.85	18.20 ± 0.75	18.40 ± 0.53	18.24 ± 2.60	<0.0001
	b*	18.87 ± 2.04	19.58 ± 2.42	20.02 ± 2.22	20.95 ± 0.84	21.09 ± 0.69	21.32 ± 1.95	<0.0001
	ITA°	35.91 ± 7.22	34.81 ± 7.34	32.89 ± 8.61	30.64 ± 2.23	29.97 ± 2.19	26.40 ± 7.62	<0.0001
	Hab°	48.19 ± 3.95	48.38 ± 3.84	48.66 ± 3.65	49.13 ± 1.52	48.96 ± 1.06	49.69 ± 3.98	0.4600
	Unevenness	33.17 ± 2.97	33.85 ± 2.78	34.27 ± 3.00	33.76 ± 1.08	34.64 ± 0.93	33.04 ± 2.59	0.0425
	Gloss	0.11 ± 0.06	0.12 ± 0.06	0.12 ± 0.06	0.10 ± 0.02	0.09 ± 0.02	0.06 ± 0.05	<0.0001
	Spots	59.22 ± 32.77	67.64 ± 39.52	91.55 ± 41.33	102.10 ± 16.27	105.20 ± 13.18	102.63 ± 44.11	<0.0001
Contour	Jaw angle	108.10 ± 5.51	107.00 ± 4.13	106.47 ± 4.77	107.21 ± 1.90	106.51 ± 1.25	109.00 ± 4.54	0.0131
	Side angle	167.85 ± 1.69	168.39 ± 1.56	168.5 ± 1.60	168.22 ± 0.64	168.38 ± 0.47	167.76 ± 1.55	0.0630
	Lower face Width	2,715.43 ± 168.56	2,661.49 ± 105.30	2,678.93 ± 121.99	2,661.91 ± 43.09	2,653.84 ± 26.46	2,711.81 ± 125.48	0.0768
	Nasolabial Angle	19.82 ± 1.30	19.92 ± 1.73	20.23 ± 1.95	20.06 ± 0.65	20.52 ± 0.64	19.31 ± 1.65	0.0069
	Nasal root Angle	62.12 ± 2.77	62.45 ± 3.89	61.29 ± 4.46	61.58 ± 1.32	60.96 ± 1.27	60.78 ± 3.51	0.1627
	Left eye width	652.37 ± 42.63	636.98 ± 21.91	641.15 ± 21.28	631.35 ± 9.72	626.47 ± 7.87	623.83 ± 28.28	<0.0001
	Right eye width	647.78 ± 42.19	630.68 ± 23.67	632.96 ± 20.82	623.48 ± 10.66	619.58 ± 7.70	620.49 ± 29.62	<0.0001
	Lip thickness	209.77 ± 14.08	205.16 ± 12.44	207.00 ± 11.97	199.41 ± 5.04	201.68 ± 4.52	194.42 ± 12.5	<0.0001
	U-area	101.89 ± 4.61	101.89 ± 5.49	102.60 ± 6.51	102.05 ± 6.06	102.12 ± 4.59	103.81 ± 5.21	0.6250
	Triangular area	31.18 ± 1.02	31.59 ± 1.28	31.82 ± 1.94	31.72 ± 1.44	31.57 ± 1.44	31.98 ± 0.93	0.0918
Texture	Coarseness	27.25 ± 0.39	27.18 ± 0.27	27.13 ± 0.30	27.00 ± 0.12	26.88 ± 0.11	26.40 ± 0.66	<0.0001
	Contrast	61.28 ± 4.73	63.63 ± 3.01	62.40 ± 3.69	62.44 ± 1.51	61.49 ± 1.23	57.49 ± 5.56	<0.0001
	Directionality	0.67 ± 0.01	0.67 ± 0.01	0.67 ± 0.01	0.67 ± 0.00	0.67 ± 0.00	0.66 ± 0.01	0.0032
	Wrinkles	2.80 ± 1.95	3.07 ± 2.12	3.43 ± 2.18	4.38 ± 0.77	4.29 ± 0.80	8.03 ± 4.11	<0.0001
	Acne	18.08 ± 15.04	14.81 ± 10.04	13.57 ± 8.87	12.81 ± 3.12	12.34 ± 1.94	12.13 ± 6.76	0.1934
	Pores	380.80 ± 393.25	344.26 ± 361.20	449.05 ± 470.29	465.00 ± 144.97	746.40 ± 225.74	1,090.16 ± 771.58	<0.0001
Porphyrin	Porphyrin	1,752.14 ± 1,715.67	1,737.43 ± 1,790.61	2,178.32 ± 2,052.93	4,589.21 ± 4,555.54	1,858.26 ± 380.53	1,748.89 ± 1,316.03	0.2716

**Figure 7:**
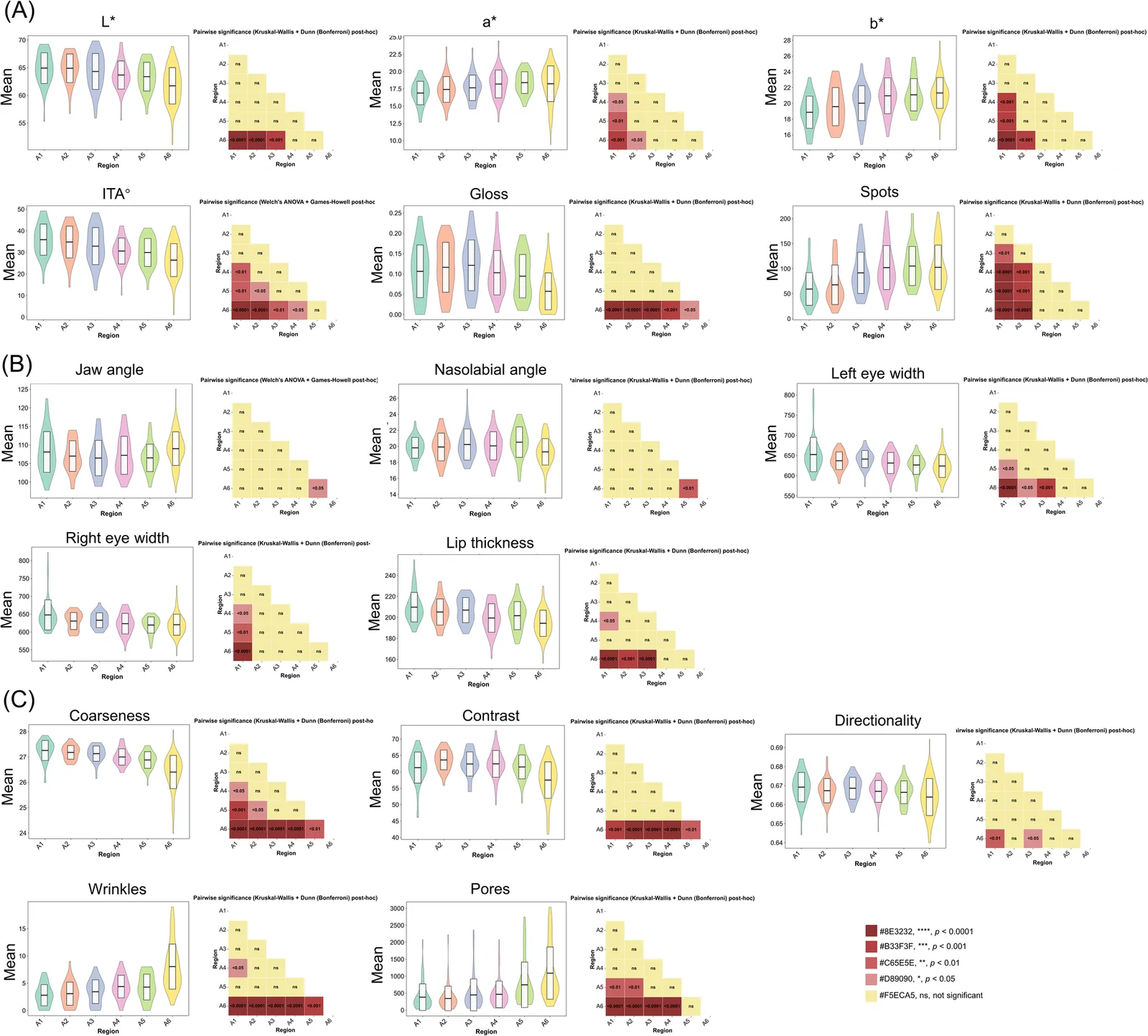
*Post hoc* multiple comparisons of facial aging indicators among the six age groups. **(A)** Tone related indicators, including L*, a*, b*, ITA°, gloss, and spots; **(B)** contour-related indicators, including jaw angle, nasolabial angle, left-eye width, right-eye width, and lip thickness; and **(C)** texture related indicators, including coarseness, contrast, directionality, wrinkles, and pores. A1–A6 represent the six age-related facial aging stages. Significant pairwise differences between age groups are indicated in the figure. *P < 0.05, **P < 0.01, ***P < 0.001, and ****P < 0.0001.

For tone indicators, L*, a*, b*, ITA°, unevenness, gloss, and spots differed significantly among the age groups, whereas no significant difference was observed for Hab°. L* and ITA° gradually decreased from A1 to A6, while a* and b* were generally higher in the older groups than in the younger groups. The number of spots increased from A1 to the middle- and older-age groups and remained relatively high in A5 and A6. Gloss showed an overall decreasing trend across the age groups, with the lowest value observed in A6. Unevenness also differed significantly among the groups, although the magnitude of variation was relatively small and did not follow a strictly monotonic pattern. These findings indicate that age-related differences in skin tone were primarily reflected in skin brightness, chromaticity, gloss, pigmentation, and complexion uniformity.

Regarding contour, significant intergroup differences were observed in jaw angle, nasolabial angle, left-eye width, right-eye width, and lip thickness, whereas side angle, lower-face width, and nasal-root angle showed no statistically significant differences. Bilateral eye width and lip thickness tended to be lower in the older groups, particularly in A5 and A6. Jaw angle and nasolabial angle also varied significantly among the groups, although their changes were less linear. Thus, age-related contour differences were mainly concentrated in eye width, lip thickness, and selected angular measurements rather than being uniformly distributed across all contour indicators.

Texture analysis showed significant differences in coarseness, contrast, directionality, wrinkles, and pores across the age groups, whereas acne showed no significant age-related variation. Wrinkle counts increased from A1 to A5 and showed a marked increase in A6. Pore counts also increased substantially in the older groups, with the highest value observed in A6. Coarseness and contrast decreased with age, whereas directionality showed only a small but statistically significant difference among the groups. These results suggest that age-related texture changes were particularly evident in wrinkles and pores, especially in the oldest age group.

No significant differences in porphyrin levels were observed among the six age groups, suggesting that porphyrin was not closely associated with chronological age in the present dataset. Overall, the intergroup comparisons demonstrated that facial aging changes were dimension-specific. Skin tone indicators reflected broad changes in brightness, chromaticity, gloss, pigmentation, and complexion uniformity; facial contour indicators showed more selective differences in eye width, lip thickness, and angular measurements; and skin texture indicators were characterized by pronounced changes in wrinkles and pores in the older age groups.

### 3.6 Performance of the deep learning-based six-class aging model

The training process and classification performance of the revised hybrid multimodal model are shown in Figure 8. As shown in Figure 8A, both training and validation losses gradually decreased during the 100-epoch training process, with the validation loss following a similar decreasing trend. Figure 8B shows the changes in Top-1 and Top-2 accuracy during training, with both metrics increasing progressively and reaching relatively stable values in the later epochs. The out-of-fold confusion matrix obtained from stratified 5-fold cross-validation (Figure 8C) illustrates the distribution of predicted and actual age stages across all 300 participants. Higher classification consistency was observed in the youngest (18–24 years) and oldest (48–60 years) groups, while misclassifications were mainly distributed between adjacent age stages, consistent with the ordered progression of facial aging.

**Figure 8:**
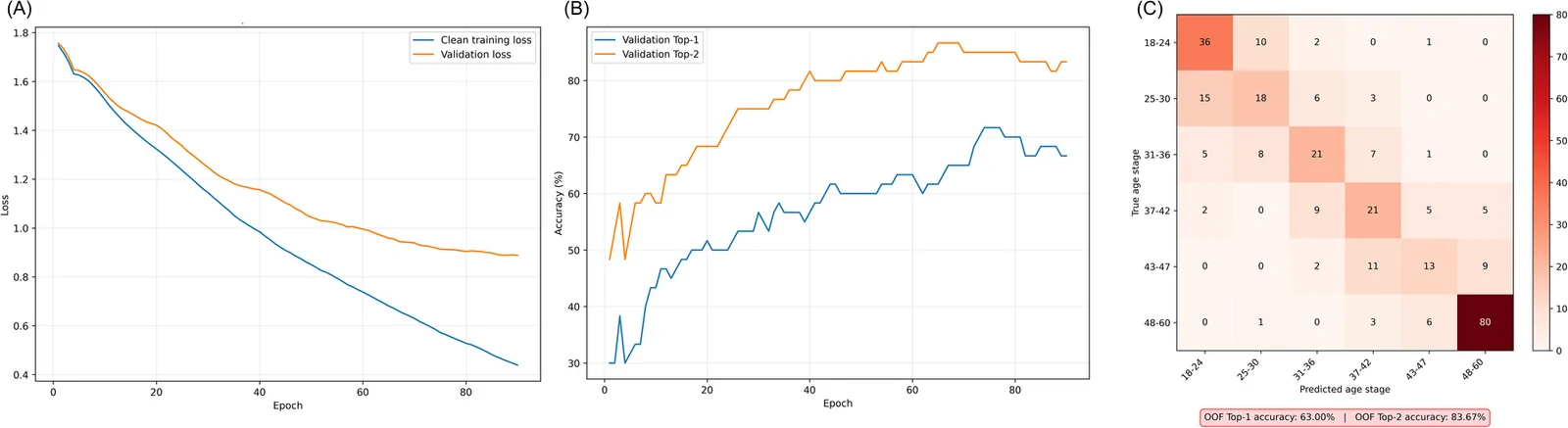
Performance evaluation of the revised hybrid multimodal facial aging classification model. **(A)** Training and validation loss curves over 100 epochs, showing the optimization process and model generalization behavior. **(B)** Training and validation Top-1 and Top-2 accuracy curves over 100 epochs. **(C)** Out-of-fold confusion matrix obtained from stratified 5-fold cross-validation across all 300 participants.

The revised model was evaluated using stratified 5-fold cross-validation with pooled out-of-fold (OOF) predictions from all 300 participants (Table 5). The model achieved an OOF Top-1 accuracy of 63.00%, with a Macro-F1 score of 0.5699 and a QWK of 0.8967. In addition, the model achieved 83.67% Top-2 accuracy and 91.67% within ±1 stage accuracy, reflecting the ordinal relationship among different facial aging stages.

**Table 5:** Performance of the multimodal facial aging classification model based on 5-fold cross-validation.

Metric	Pooled OOF	95% CI	5-fold Mean ± SD
Top-1 Accuracy	63.00%	58.00%–67.67%	63.00% ± 6.81%
Macro-F1	0.5699	0.5107–0.6251	0.5606 ± 0.0874
QWK	0.8967	0.8664–0.9216	0.9001 ± 0.0669
Top-2 Accuracy	83.67%	79.33%–87.67%	83.67% ± 5.94%
Within ±1 stage	91.67%	88.67%–94.67%	91.67% ± 8.42%

### 3.7 Explainability analysis of the hybrid multimodal facial aging classification model

In younger participants, the model primarily focused on the nasal and perioral regions, which may reflect early differences in facial appearance characteristics among younger age stages. In older participants, attention was increasingly observed around the periorbital region, consistent with the prominent age-related changes commonly occurring in this area (Figure 9).

**Figure 9:**
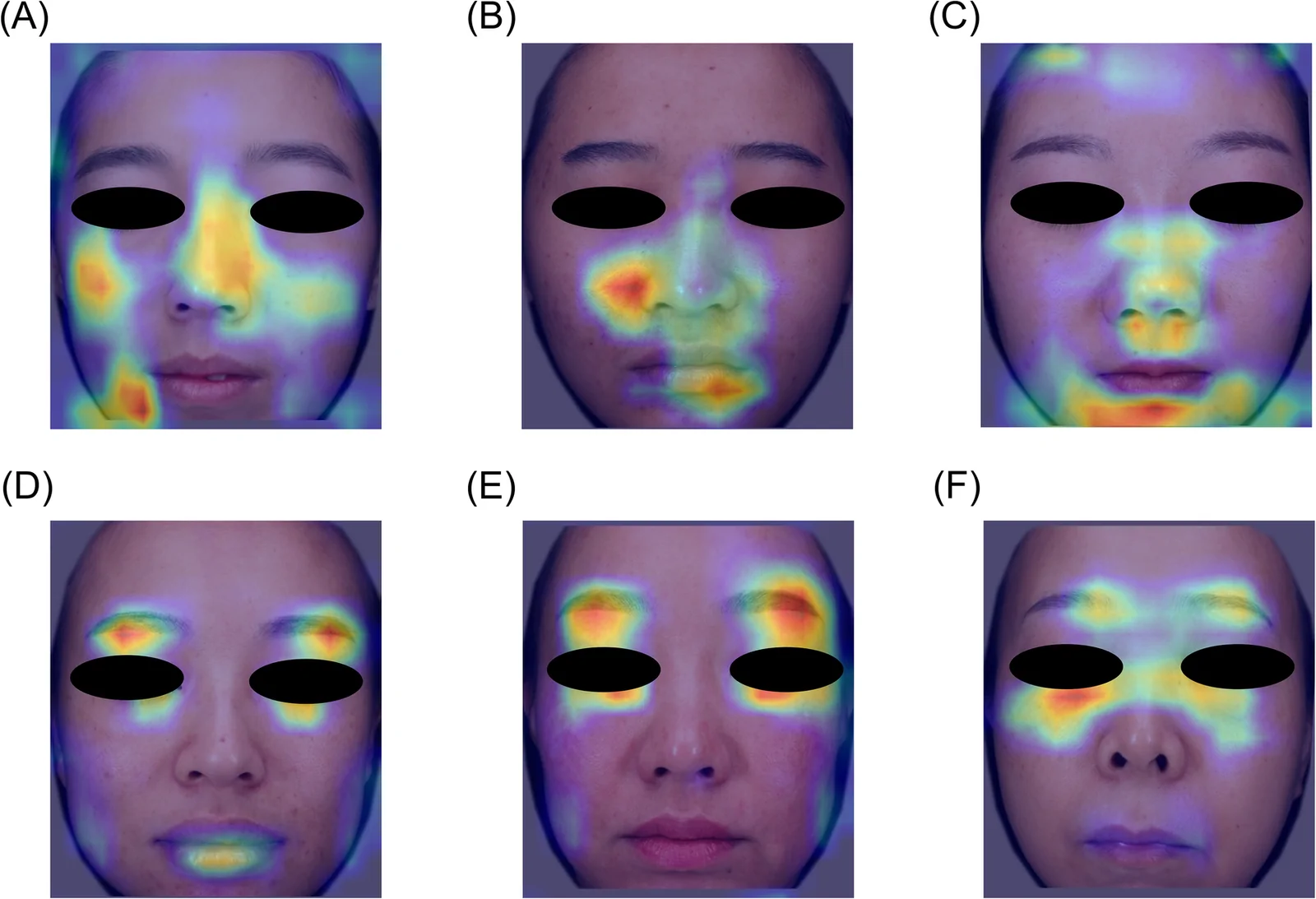
Grad-CAM visualization of facial regions contributing to age-stage classification. **(A)** A1; **(B)** A2; **(C)** A3; **(D)** A4; **(E)** A5; **(F)** A6.

SHAP-based feature importance analysis was performed to identify the major indicators contributing to the classification of each facial aging stage. The top 20 ranked features showed clear stage-specific differences (Figure 10), and the five most influential indicators for each stage are summarized in Table 6.

**Figure 10:**
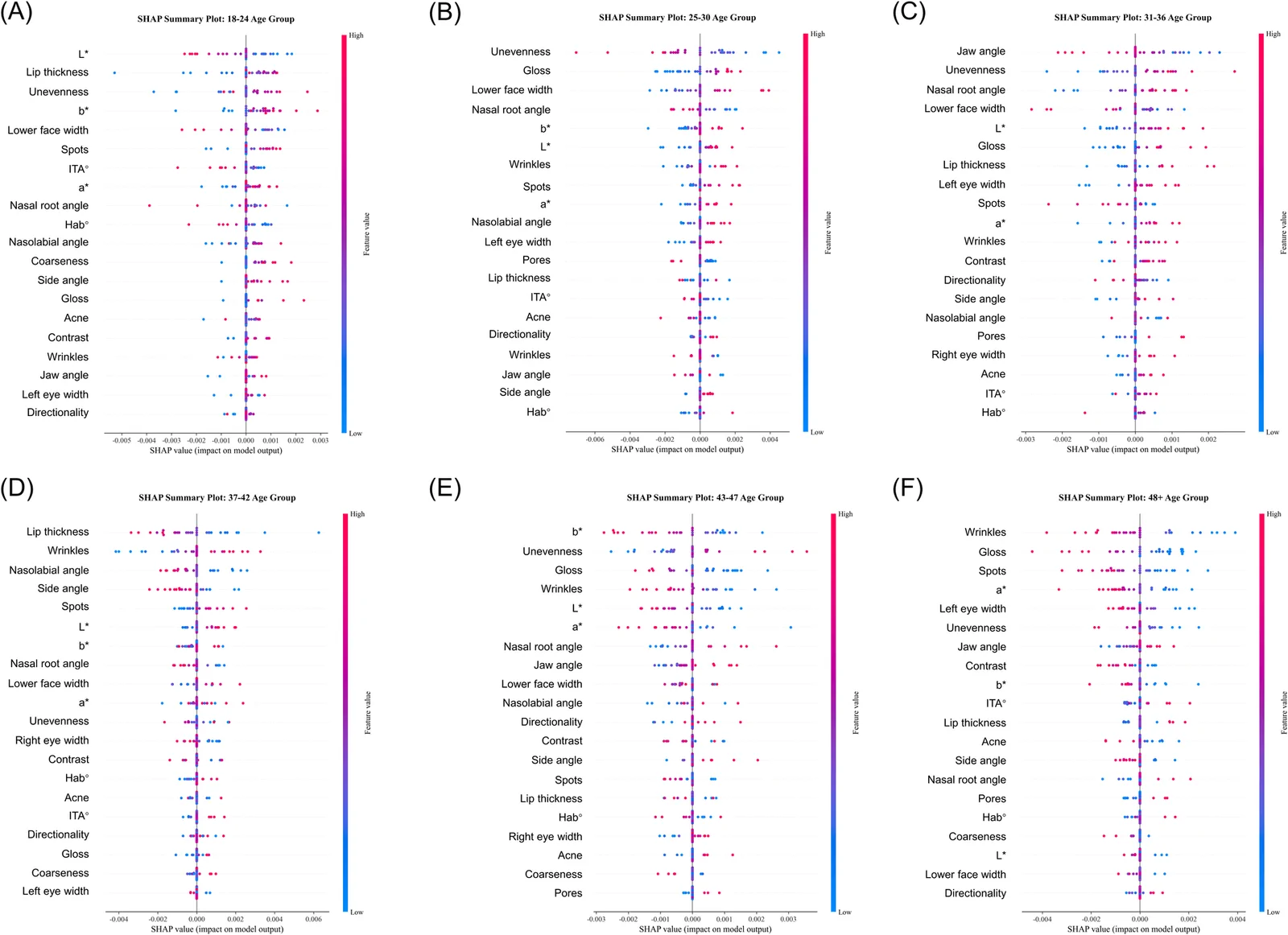
SHAP-based feature importance analysis of the hybrid multimodal facial aging classification model. **(A)** A1: High-Translucence Period, 18–24 years; **(B)** A2: Complexion Fluctuation Period, 25–30 years; **(C)** A3: Contour Initiation Period, 31–36 years; **(D)** A4: Contour Loosening Period, 37–42 years; **(E)** A5: Pigmentation Emergence Period, 43–47 years; and **(F)** A6: Texture Reinforcement Period, 48-60 years.

**Table 6:** Top five facial aging indicators ranked by SHAP contribution across different aging stages.

Group	Rank 1	Rank 2	Rank 3	Rank 4	Rank 5
A1	L*	Lip thickness	Unevenness	b*	Lower face width
A2	Unevenness	Gloss	Lower face width	Nasal root angle	b*
A3	Jaw angle	Unevenness	Nasal root angle	Lower face width	L*
A4	Lip thickness	Wrinkles	Nasolabial angle	Side angle	Spots
A5	b*	Unevenness	Gloss	Wrinkles	L*
A6	Wrinkles	Gloss	Spots	a*	Left eye width

The stage-dependent distribution of SHAP-ranked indicators suggests a sequential phenotypic transition during facial aging, progressing from changes in skin tone and complexion to facial contour remodeling and, ultimately, to texture deterioration (Figure 11). During the early aging stages, the model relied primarily on indicators of skin tone and complexion uniformity, suggesting that subtle changes in skin brightness, chromaticity, pigmentation, and unevenness may represent the earliest detectable features of facial aging. As aging progressed to the intermediate stages, contour variables contributed more substantially to classification, indicating that facial structural remodeling gradually became more important for distinguishing among aging stages. In the later stages, wrinkles, reduced gloss, pigmentation features, and texture indicators emerged as the dominant contributors, suggesting that visible textural deterioration and other aging signs were the most prominent characteristics of advanced facial aging. Overall, the SHAP results revealed an interpretable trajectory in which facial aging progressed from changes in skin tone and complexion to contour remodeling, followed by wrinkle- and texture-dominated aging patterns.

**Figure 11:**
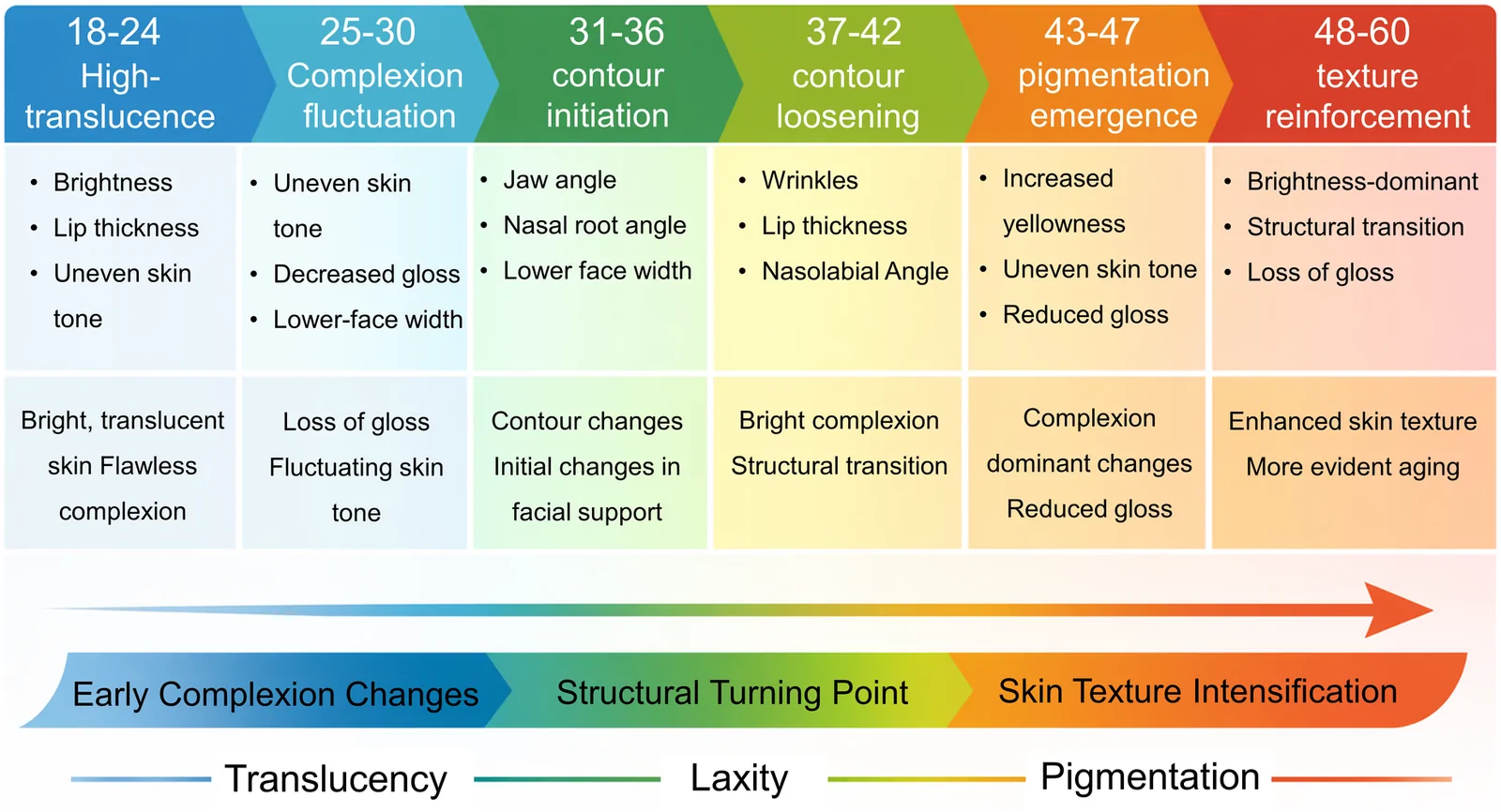
Age-related progression of facial aging characteristics from complexion changes to facial contour alterations and skin texture intensification across six age groups.

## 4 Discussion

Facial aging is a progressive and multidimensional process involving coordinated changes in skin appearance, facial morphology, and surface texture. In this study, quantitative facial phenotyping, deep learning-based classification, and SHAP interpretation consistently revealed an age-associated shift in the relative prominence of different facial aging features in Chinese women. Although complexion, contour, and texture changes may overlap across the aging process, complexion-related alterations were more prominent in the earlier stages, contour remodeling became increasingly evident in the intermediate stages, and textural deterioration predominated in the advanced stages. This pattern was supported by age-related feature fitting, comparisons across six aging groups, and explainable model analysis, suggesting that facial aging comprises distinct but interconnected phenotypic phases rather than a uniform accumulation of aging signs.

Facial aging is influenced by intrinsic biological processes and extrinsic exposome-related factors, including ultraviolet exposure and environmental stress (27). Recent studies have further highlighted the central role of dermal fibroblast senescence, extracellular matrix remodeling, and altered cellular-microenvironment interactions in driving age-related skin changes (4, 5, 28). These biological alterations are increasingly recognized as major contributors to the visible changes in skin texture, pigmentation, and facial morphology observed during aging. Ethnic differences have also been reported, with Asian populations, particularly Chinese women, exhibiting relatively prominent pigmentation changes and distinctive wrinkle-development patterns (9, 10, 29). Population-specific assessment is therefore necessary to accurately characterize facial aging. By integrating objective facial measurements with interpretable artificial intelligence, the present study provides a quantitative framework for examining age-associated changes in Chinese women and for identifying the dominant phenotypic characteristics of different aging stages.

In the earlier aging stages, changes in tone and complexion uniformity were the most prominent features. Multiple color-related parameters, including L*, a*, b*, ITA°, unevenness, gloss, and spots, differed significantly among the six aging groups (Figure 7), while age-related fitting demonstrated progressive changes in skin brightness, pigmentation distribution, and optical properties (Figure 6). These findings indicate that alterations in facial appearance can be detected before pronounced morphological and textural deterioration becomes dominant. Recent AI-assisted aging simulation studies have also highlighted hyperpigmentation as an early and prominent facial aging marker in Asian populations, particularly under different UV exposure scenarios (30). Previous objective colorimetric studies have shown that CIELAB-derived parameters, particularly ITA°, are useful for evaluating age-associated variation in skin brightness and pigmentation (31, 32). Recent studies using multidimensional color assessment approaches have further demonstrated that brightness, chromaticity, and color uniformity jointly contribute to objective evaluation of Chinese women's skin characteristics (23, 33). Skin color distribution and homogeneity have also been associated with perceived facial age and skin health (34, 35). Consistent with these observations, our SHAP analysis showed that color-tone and complexion-uniformity indicators made greater contributions to classification in the younger aging stages (Figure 10), supporting their importance as early aging-related phenotypes.

With increasing age, contour remodeling became more evident and appeared to represent an intermediate phase between early complexion changes and advanced textural deterioration. Jaw angle, nasolabial angle, eye width, and lip thickness showed significant differences among the six aging groups (Figure 7), indicating age-associated changes in facial geometry and proportions. The UFI model, based on the U-frame and triangular regions, also showed continuous age-related trajectories in facial geometric characteristics (Figure 5). Although UFI parameters did not differ significantly among the predefined groups, their fitted trends suggested gradual morphological changes that may not be fully captured by categorical comparisons. Previous studies have described facial aging as an “inside-out” process involving coordinated changes in skeletal structures, fat compartments, muscles, and soft tissues (24). Remodeling of the mandible and midface can affect facial support, jawline definition, and overall proportions (36, 37), while three-dimensional facial analyses have identified age-associated changes in eye dimensions, facial width, and lip morphology (26, 38). Together, these findings support contour remodeling as an important transitional component of facial aging.

In the advanced aging stages, deterioration of texture became the predominant phenotypic characteristic. Large-scale AI-based analyses in Chinese populations have recently confirmed that pore enlargement exhibits age-dependent patterns and is influenced by environmental factors, further supporting the importance of texture-related parameters in population-level facial aging assessment (39). Wrinkles, pores, coarseness, contrast, and directionality differed significantly among the aging groups (Figure 7), and their fitted trajectories demonstrated progressive deterioration in skin-surface characteristics with increasing age (Figure 6). SHAP analysis further identified wrinkles, pores, reduced gloss, pigmentation-related indicators, and texture parameters as major contributors to advanced-stage classification (Figure 10). Previous studies have shown that image-derived texture descriptors can reflect changes in skin-surface heterogeneity and microtopography (40, 41), and studies of Chinese women have reported progressive increases in wrinkles, pores, and other visible surface-aging signs with age (22, 23). Our results extend these findings by showing that texture-related indicators become increasingly influential in distinguishing advanced aging stages, marking a shift from predominantly complexion- and contour-related changes toward more visible surface deterioration.

The deep learning model further demonstrated that these multidimensional aging phenotypes could be effectively integrated for six-group classification. Its classification performance (Figure 8) indicates that the combined information contained in color, contour, and texture characteristics is sufficient to distinguish different aging stages. Previous dermatological artificial intelligence studies have shown that deep learning can extract clinically meaningful information from facial or skin images for disease classification, age prediction, and the assessment of individual aging signs (13, 42–48). Recent advances in facial age estimation have further highlighted the importance of developing interpretable and biologically meaningful AI frameworks rather than relying solely on predictive accuracy (49). However, most studies have focused on predictive performance or isolated aging manifestations rather than the stage-dependent organization of multiple phenotypic domains. The present study therefore extends conventional age-estimation approaches by using quantitative facial indicators to characterize broader patterns of facial aging progression.

An important feature of the present model is the use of SHAP analysis to improve the phenotypic interpretability of its predictions. The stage-specific SHAP distributions showed a progressive shift in the features most strongly associated with model classification: color-tone and complexion-uniformity indicators contributed more prominently in the earlier stages, contour-related variables became more influential in the intermediate stages, and wrinkles, pores, and texture-associated indicators predominated in the advanced stages (Figure 10). Explainable artificial intelligence has increasingly been applied in medical image analysis to clarify the contribution of individual variables to model predictions, and SHAP-based approaches have been used to identify relevant phenotypic features in dermatological applications (50–53). Compared with models focused primarily on predictive accuracy, our framework links classification outcomes with measurable facial characteristics and reveals stage-dependent aging patterns. This approach aligns with recent advances in explainable multimodal AI, where integrating heterogeneous data with interpretable methods improves the understanding of model predictions (21).

Based on these findings, we developed a six-stage facial aging framework that can be summarized into three broader phenotypic phases: an earlier complexion-dominant phase, an intermediate contour-remodeling phase, and an advanced texture-dominant phase (Figure 10). Unlike systems based solely on chronological age, this framework incorporates quantitative facial characteristics and interpretable model outputs to describe differences in the relative prominence of aging phenotypes. Previous studies have proposed aging classifications for Chinese women based on multiple facial characteristics and have demonstrated continuous age-associated phenotypic changes (19, 20). Our findings further integrate these characteristics into an interpretable progression framework that may support objective facial aging assessment and personalized skincare evaluation. Nevertheless, the cross-sectional design does not directly establish within-individual temporal transitions, and longitudinal studies are required to verify the proposed sequence. Future studies incorporating lifestyle factors, environmental exposure, molecular information, and multicenter populations may further clarify the mechanisms and population variability underlying facial aging.

Several lifestyle and physiological factors, such as smoking status, skincare practices, and hormonal status, may influence facial aging trajectories through oxidative stress, inflammation, barrier function regulation, and collagen metabolism. These variables were not systematically collected or incorporated into the present analysis, which may represent potential confounding factors affecting individual variations in facial aging phenotypes. Future studies incorporating comprehensive lifestyle information and physiological assessments may further improve the accuracy and interpretability of facial aging evaluation. In addition, the relatively limited sample size of the present study may restrict the generalizability of the findings, and future investigations involving larger and more diverse populations are warranted to validate and extend the current results.

## 5 Conclusion

This study provides a quantitative and interpretable characterization of facial aging in Chinese women by integrating objective facial phenotyping, deep learning classification, and SHAP analysis. Facial aging was not manifested as a uniform accumulation of visible signs but showed a stage-dependent shift in the relative prominence of different phenotypic domains. Complexion-related alterations were more prominent in the earlier stages, facial contour remodeling became increasingly evident during the intermediate stages, and wrinkles, pores, and other texture-related changes predominated in the advanced stages. Based on these findings, we proposed a six-stage facial aging framework encompassing three broader phenotypic phases: a complexion-dominant phase, a contour-remodeling phase, and a texture-dominant phase. Future studies with larger, multicenter, and more diverse populations are needed to further optimize the classification model, improve its robustness and generalizability, and validate the proposed aging framework across different populations and imaging conditions.

## Data Availability

The raw data supporting the conclusions of this article will be made available by the authors, without undue reservation.
