# Supplementary material for: Explainable deep learning reveals a stagewise temporal deterioration from skin tone to contour to texture in Chinese women’s skin aging
Source: Front Med (Lausanne). 2026 Sep 25;13:1945376. doi: 10.3389/fmed.2026.1945376 (PMC13649388; doi:10.3389/fmed.2026.1945376)
Supplement: Supplementary file 1 [file Table1.pdf]

Table S1 Comparison of linear, quadratic, and cubic polynomial models for age-related facial indicator changes

Indicator	Linear R^2	Quadratic R^2	Cubic R^2	Cubic adjusted R^2	Linear AIC	Quadratic AIC	Cubic AIC	Linear BIC	Quadratic BIC	Cubic BIC	Cubic β3 P value
L*	0.48	0.49	0.56	0.53	21.41	23.28	18.53	24.93	28.56	25.58	0.01
a*	0.14	0.35	0.44	0.39	4.00	-6.06	-10.31	7.52	-0.77	-3.27	0.02
b*	0.61	0.68	0.69	0.66	-26.02	-32.51	-31.21	-22.49	-27.23	-24.16	0.43
ITA°	0.59	0.60	0.65	0.62	94.42	95.84	92.07	97.94	101.12	99.11	0.02
Hab°	0.20	0.26	0.31	0.27	30.91	29.45	27.40	34.43	34.73	34.44	0.06
Unevenness	0.01	0.21	0.22	0.16	29.49	21.70	23.54	33.02	26.99	30.59	0.71
Gloss	0.43	0.52	0.61	0.57	1059.33	1053.49	1048.05	1062.86	1058.78	1055.10	0.01
Spots	0.47	0.62	0.65	0.62	244.70	232.85	231.92	248.22	238.13	238.97	0.11
Jaw angle	0.07	0.25	0.26	0.20	67.76	60.30	61.97	71.28	65.59	69.01	0.58
Side angle	0.04	0.19	0.21	0.14	-29.28	-34.61	-33.26	-25.76	-29.32	-26.21	0.44
Lower face width	0.01	0.19	0.23	0.17	358.06	351.47	351.16	361.58	356.75	358.20	0.15
Nasolabial angle	0.09	0.26	0.27	0.21	-32.18	-38.90	-37.62	-28.66	-33.62	-30.57	0.42
Nasal root angle	0.13	0.13	0.14	0.07	43.59	45.57	47.05	47.12	50.85	54.10	0.50
Eye width left	0.41	0.46	0.5	0.46	215.93	214.80	213.20	219.45	220.08	220.24	0.07
Eye width right	0.38	0.50	0.53	0.50	213.58	206.66	205.53	217.11	211.95	212.57	0.09
Lip	0.53	0.53	0.54	0.50	204.91	206.90	208.12	208.43	212.19	215.17	0.40
Coarseness	0.70	0.81	0.81	0.80	-125.10	-142.49	-141.21	-121.58	-137.21	-134.16	0.42
Contrast	0.42	0.69	0.71	0.68	74.05	49.13	48.45	77.57	54.42	55.50	0.12
Directionality	0.28	0.28	0.28	0.23	-495.06	-493.24	-491.25	-491.53	-487.95	-484.21	0.91
Wrinkles	0.66	0.79	0.82	0.80	40.30	22.61	19.58	43.82	27.89	26.63	0.03
Acne	0.28	0.33	0.37	0.32	102.66	101.65	101.22	106.18	106.94	108.27	0.14
Pores	0.60	0.72	0.73	0.70	477.83	463.75	465.65	481.35	469.04	472.69	0.76
Porphyrin	0.01	0.09	0.10	0.02	553.82	552.06	553.96	557.34	557.34	561.00	0.77

Table S2 Estimated stationary and inflection points of cubic polynomial models within the studied age range (18–60 years)

Indicator	Stationary point	95% CI of stationary point	Inflection point	95% CI of inflection point
L*	25.55; 53.46	19.98-28.34; 50.61-58.57	39.50	36.59-42.63
a*	20.38; 46.55	18.44-47.10; 45.29-48.81	33.47	25.44-36.44
b*	50.56	18.90-57.78	29.40	20.24-55.91
ITA°	22.70; 52.80	18.42-56.62; 50.27-57.20	37.75	30.84-40.68
Hab°	29.82; 41.13	21.32-38.17; 35.49-46.66	35.48	23.93-40.05
Unevenness	36.42	23.22-41.57	—	—
Gloss	28.70; 57.40	24.45-30.72; 52.97-59.83	43.10	40.38-50.85
Spots	48.36	18.38-51.77	31.16	21.13-35.93
Jaw angle	34.00	23.24-39.72	59.40	19.92-58.71
Side angle	33.60	26.13-39.34	51.70	20.63-58.21
Lower face width	33.60; 59.30	30.10-38.33; 50.54-59.68	46.50	28.99-56.81
Nasolabial angle	38.40	19.16-40.71	25.60	19.40-54.84
Nasal root angle	24.50; 55.20	19.18-54.05; 47.67-58.86	39.80	27.17-51.29
Left eye width	—	—	42.51	33.37-50.23
Right eye width	43.83; 47.29	34.17-53.48; 44.11-58.12	45.56	28.50-56.50
Lip thickness	—	—	39.41	26.27-51.15
Coarseness	—	—	—	—
Contrast	31.10	28.71-33.54	52.80	27.18-58.59
Directionality	—	—	26.53	26.27-53.42
Wrinkles	—	—	30.40	21.90-34.31
Acne	40.99; 47.67	—	44.33	30.07-51.96
Pore	27.30	21.26-30.97	—	—
Porphyrin	35.77	19.61-43.68	—	—
